# British elite swimmers’ experiences and perspectives on life skill development

**DOI:** 10.3389/fpsyg.2024.1344352

**Published:** 2024-03-22

**Authors:** Ross Murdoch, Hee Jung Hong

**Affiliations:** Faculty of Health Sciences and Sport, University of Stirling, Stirling, Scotland, United Kingdom

**Keywords:** athletic career, career development, career transition, elite swimming, transferrable skills

## Abstract

This study explores the experiences of British elite swimmers in developing life skills during and throughout their athletic careers, examining the factors that influence their perspectives on this skill development. Six high-profile British swimmers, who have competed at the Commonwealth and/or Olympic Games, were recruited for this study. Semi-structured interviews were conducted, and thematic analysis was applied. Through the analysis, two key themes were identified from the thematic analysis: (a) Implicit life skill development through athletic and educational experience, and (b) Understanding the influence of swimming on life skill development. The first theme includes three sub-themes: (a) Establishment of athletic identity, (b) Prioritization of athletic identity, and (c) Navigating life skills through athletic challenges. The findings show that the swimming careers of participants and their associated identities contributed to the development of a wide range of implicit life skills. This growth was facilitated by both educational and sporting experiences, with all participants reporting positive personal development from their time in competitive swimming. The findings in this study enhance our understanding of life skill development and provide insights into how to more effectively support high-performance athletes in both their athletic careers and educational endeavors.

## 1 Introduction

The development of life skills through sports participation has long been a subject of interest among researchers in the field of sport psychology (Pierce et al., 2017). Life skills are defined as “those internal personal assets, characteristics, and skills such as goal setting, emotional control, self-esteem, and hard work ethic that can be facilitated or developed in sport and transferred for use in non-sport settings” (Gould and Carson, 2008, p. 60). UNICEF (2003) also describes life skills as “psychosocial abilities for adaptive and positive behavior that enable individuals to deal effectively with the demands and challenges of everyday life” (para. 3). Indeed, for elite athletes, life skills developed in sports are crucial for excelling in competitive environments and managing life beyond their athletic careers (de Subijana et al., 2022). The widespread belief among sports practitioners is that skills such as leadership and teamwork, nurtured through sports participation, as a matter of course prepare athletes for success in other life domains (Trottier and Robitaille, 2014). Sports organizations often highlight the belief that participating in sports develops life skills crucial for societal and personal growth (Pierce et al., 2017). This indicates that life skills developed in sports are beneficial in other areas of life. However, such perspective has been also challenged by some researchers (e.g., Coakley, 2011) that question the actual learning of these skills through sports and their applicability to non-sporting situations. On the other hand, researchers have studied positive youth development (PYD) and life skills in sport, which advocate life skills development via sport. PYD and life skills have become significant to exploring psychosocial evolution in the context of youth sports studies (Camiré et al., 2022). In this context, life skills are frequently identified as crucial developmental outcomes, highlighting their importance in the growth of youth as Holt et al. (2017) pointed out, “life skill building activities are an essential feature of programs designed to foster PYD” (p.3). Holt et al. (2017) also suggested that integrating an indirect method such as developing PYD environment into a direct strategy including focusing on a life skills program can result in positive effects, which can be more likely to happen in sports setting that are well-organized and guided by skilled and supportive adults (Camiré et al., 2022).

Despite considerable research over the past decade focusing on life skills development in sports (e.g., Gould et al., 2007; Holt et al., 2008), there is a notable scarcity of studies that explicitly explore the transfer of life skills. Martinek and Lee (2012) highlighted the gap in our understanding of how life skills are transferred in the context of sports. In this context, the research by Jones and Lavallee (2009) involved a detailed study of a former top-tier tennis athlete, focusing on her use of communication skills and self-assurance acquired during her sports career in her educational environment. In a related study, Camiré et al. (2012) investigated the perceptions of life skills transfer among high school coaches and athletes, finding a consensus that such skills, developed in the athletic realm, were indeed being applied by the athletes in other areas of their lives. While the previous studies offer insights into the development and transfer of life skills from athletic careers to non-athletic domains, much of this research centers on youth populations. Investigations specifically targeting high-performance athletes, such as Olympians, are still limited. While research on high-performance athletes’ development of life skills through sport is under-developed, de Subijana et al. (2022) explored the perspectives of retired high-performance athletes regarding their life skill development in sports. In a cross-sectional study involving 477 former elite athletes who completed a questionnaire, it was found that athletes with higher education levels at the time of their retirement reported possessing more advanced individual and social life skills. Specifically, athletes from team sports and those who trained less than 27 h per week perceived themselves as having superior social skills. Age was also a factor, with older athletes indicating a higher proficiency in social life skills. In addition, athletes with higher monthly salaries felt more confident in their individual and social life skills (de Subijana et al., 2022). These findings imply that sports stakeholders should provide life skills courses to athletes and guide them on how to transfer these skills to life after sports.

In respect of high-performance career and life after competitive sports, athletic identity plays a significant role. Identity is viewed as a complex and evolving concept, shaped by various stable yet socially influenced dimensions (Markus, 1977; Stryker, 1978; Stryker and Serpe, 1994). In sports context, athletic identity refers to the degree to which a person identifies with the role of an athlete and sees themselves in this role. Individuals with a strong athletic identity tend to view their experiences through the lens of an athlete (Lally, 2007). Often, one aspect of an individual’s identity, such as athlete identity, becomes dominant, overshadowing others. This focus on a single identity aspect can lead to the neglect of other potential roles, potentially causing future identity challenges due to this imbalance (Lally, 2007). In late adolescence, the key developmental challenge is forming a personal identity (Erikson, 1959). While developmental theorists note that diverse experiences and social interactions are key to this process (Jordaan, 1963; Super, 1990), athletes often focus intensely on sports, missing broader exploratory activities, which can hinder their self-identity development (Brown et al., 2000). This limited exploration can lead to what is known as identity foreclosure, where athletes overly identify with their sport to the exclusion of other identities (Petitpas and Champagne, 1988; Pearson and Petitpas, 1990). *Identity foreclosure*, a term first introduced by Erikson (1959), was later detailed by Marcia (1966) as part of adolescent ego-identity development. It describes the premature commitment to roles and ideologies that align with social or parental expectations, often to avoid identity crises. This commitment can offer psychological security but restricts personal freedom and psychosocial growth. Marcia (1966) proposed that the best development of ego-identity comes from exploring diverse possibilities and making conscious, well-informed choices. Individuals in identity foreclosure have not engaged in such exploration but show commitment to specific life roles (Brewer and Petitpas, 2017) such as high-performance athletes.

High-performance sports and career transitions post-athletic life have been widely studied in recent decades. This research covers various phases of an athlete’s career, from shifts in training locations and moving from school to higher education, to transitioning from junior to senior competition levels and retirement (Park et al., 2013; Demetriou et al., 2018; Stambulova et al., 2021). In this context, considerable attention has been directed towards researching and understanding the consequences of retirement from competitive sports for athletes. In particular, the issue of identity reformation post-retirement has been a recurring theme in the literature, highlighting the intricate process athletes experience in redefining their sense of self beyond their sporting careers (Lally, 2007; Park et al., 2013). Torregrosa et al. (2015) highlighted a key indicator of potential issues post-retirement for elite athletes is their exclusive focus on their sport, resulting in a strong, one-dimensional athletic identity. In their study of qualitative longitudinal study that examined the retirement process of Olympians, they pointed out that elite athletes with a linear trajectory, focusing solely on their sporting career (Pallarés et al., 2011), are often ill-prepared for retirement and face involuntary career termination. This might be due to a lack of social support and a tendency to use reactive coping strategies rather than proactive ones, making their transition more challenging. Park et al. (2013) conducted a systematic review of literature consisting of 122 papers that identified 15 factors that relate to the quality of career transitions. These factors included athletic identity, demographics, voluntary retirement, health issues, career and personal growth, achievements in sports, education and financial status, self-perception, life control, disengagement, time elapsed post-retirement, coach-athlete relationship, life changes, and life balance. In total, 35 studies established a connection between athletic identity and the nature of athletes’ career transitions. Of these, 34 studies found a correlation between a strong athletic identity and a high tendency toward identity foreclosure with poorer transition outcomes (Park et al., 2013). The findings indicated that athletes with a pronounced athletic identity at the time of ending their sports career often experienced a sense of identity loss (e.g., Kerr and Dacyshyn, 2000; Lally, 2007) and required a longer duration to adjust to life after sports (e.g., Grove et al., 1997; Warriner and Lavallee, 2008). In addition, a significant number of the reviewed studies (86 in total) discovered that participants faced challenges and negative feelings during their transition from sports careers. These experiences encompassed feelings of loss, identity crises, and distress associated with concluding their athletic careers and adapting to life beyond sports. Cosh et al. (2015) also suggested that athletes experiencing career transitions often faced challenges such as difficulty integrating into new jobs, anxiety about career uncertainties, and symptoms of body dysmorphia due to changes in personal appearance after sport. They propose that mitigating these negative aspects, especially during retirement from elite sport, can be achieved through social support and a well-planned transition to an alternate career. Control is also regarded as crucial at the retirement stage, as Park et al. (2013) emphasize, to facilitate a smoother transition to life after sport. In this respect, developing and applying life skills can be key in controlling and reducing the adverse effects commonly experienced during this transition.

Focusing specifically on life skills development, Demetriou et al. (2018) studied the negative career transition of an Australian rules footballer, highlighting the challenges faced when retirement is forced by external factors, such as injury. This case study showed that inadequate confidence in communication skills led to a protracted transition period and poor life choices, including alcohol abuse and family neglect (Cosh et al., 2015). It suggests that empowering elite athletes with confidence in skills acquired through sport is critical for a successful transition from sport and better integration into society, irrespective of the reasons for retirement. Stambulova et al. (2021) examined the evolution of athletic career transition research, focusing on dual-career athletes who balance sports with education or work. They suggested that managing sport, educational or work commitments, and social lives equips dual-career athletes with valuable personal resources. These include skills in “dual-career management, career planning, mental toughness, social intelligence, and adaptability” (Stambulova et al., 2021, p.531), which align with the life skills definitions. This implies that engaging in a dual career can impart essential skills transferable to various life aspects. In this regard, it is beneficial for athletes to apply the skills they possess to pre-retirement planning, as this helps them anticipate potential post-retirement issues. Furthermore, the level of satisfaction with their achievements during their athletic careers can predict the extent of challenges they may face in areas such as social networks, leisure, and finance (Barriopedro et al., 2019). While these findings highlight the importance of considering athletes’ skills in supporting elite athletes through their transition out of sports, it has been under-researched how elite athletes have developed and perceive their life skills, which are likely to be advantageous for planning or navigating their post-retirement lives. Given the findings and gap in literature, the present study explores the experiences of British elite swimmers in developing life skills during and throughout their athletic careers, examining the factors that influence their perspectives on this skill development.

## 2 Materials and methods

### 2.1 Design

This study adopted an intrinsic case study design to gain in-depth insight into the experiences of British elite swimmers in developing life skills during and throughout their athletic careers. “An intrinsic case study is typically undertaken to learn about a unique phenomenon. The researcher should define the uniqueness of the phenomenon, which distinguishes it from all others” (Crowe et al., 2011, p.1–2). This approach enabled us to conduct an in-depth examination of a particular case by concentrating on the individual experiences of those involved. Since our research aimed to deeply understand participants’ insights into their experiences, an interpretive phenomenological approach was regarded as appropriate. This approach is rooted in an interpretivist paradigm, and it is in line with relativist ontology and subjectivist epistemology (Mallett and Tinning, 2014). This philosophical paradigm allowed us to examine how each individual perceives and interprets their own experiences (Sparkes, 1992; Mallett and Tinning, 2014). Interpretive phenomenology is about describing, understanding, and interpreting phenomena to grasp the core of lived experiences (Creswell, 2007; Tuohy et al., 2013). To capture the depth of participants’ personal experiences, semi-structured interviews were conducted (McArdle et al., 2012).

### 2.2 Participants

Six elite swimmers, who have competed in the Commonwealth Games and/or Olympic Games, were recruited for the study. Convenience sampling was utilized, as the lead author had access to a suitable sample group that met the study criteria. The lead author met all the participants in person for a brief initial consultation about their participation in the study. Prior to any interviews, all participants were provided with an information sheet detailing the study and a consent form. At both of these stages, participants were informed that their involvement was completely voluntary, and they could withdraw at any time without any repercussions. The participants were aged between 21 and 33 years old at the time of the interviews (*M* = 26.5; SD = 4.14). The group comprised an equal number of female (*n* = 3) and male (*n* = 3) swimmers, with one retired competitor from each gender category (see Table 1). Five participants had competed in one or more Olympic Games, while one had participated in a Commonwealth Games. Their competitive swimming careers ranged from a minimum of 11 years to a maximum of 20 years at the time of the interviews (*M* = 16.17; SD = 3.25). These athletes reached their highest competition levels at various stages in their careers, with ages ranging between 19 and 24 years (*M* = 21.17; SD = 2.32). Within those participants, five individuals balanced their athletic careers with academic pursuits up to the undergraduate level, whereas one dedicated themselves entirely to swimming, free from any additional obligations. In addition, those swimmers have trained at one of the most high-profile swimming training centers in the U.K., where they could prepare for international competitions.

**Table 1:** Participant information.

Participant	Age/Age highest level achieved	Gender	Active or retired	If retired, No. of years retired	Years competitive	Highest level of competition (No. of editions)
P1	30/19	Female	Retired	1	19	Olympic Games (3)
P2	22/21	Female	Active	-	11	Olympic Games (1)
P3	25/19	Male	Active	-	15	Olympic Games (2)
P4	25/24	Female	Active	-	17	Olympic Games (1)
P5	33/20	Male	Retired	6	20	Olympic Games (3)
P6	24/24	Male	Active	-	15	Commonwealth Games (1)

### 2.3 Data collection

A total of six interviews were conducted with an average duration of 62 min and a range of 51 to 69 min. All interviews were semi-structured allowing for emergent data stemming from personal experiences and anecdotes. Utilizing semi-structured interviews, with open-ended questions, allowed for the subtleties of each interviewees experience to be captured and recorded (Smith and Sparkes, 2016). The personal experiences and anecdotes from each of the participants give the data the rawness that the authors wanted to capture. The semi-structured interview questions were shared with the participants in advance so that they have the opportunity to review and make a decision on which questions they were comfortable answering align with ethical considerations. The lead author conducted all interviews via Teams meeting, which were recorded and transcribed verbatim. With the semi-structured nature of the interviews, we maintained flexibility, allowing participants to share meaningful experiences that were not addressed in the interview guide (McArdle et al., 2012). However, to ensure consistency across interviews, an interview guide was established, drawing from our research questions and existing literature (e.g., Lally, 2007; Park et al., 2013; Trottier and Robitaille, 2014; Pierce et al., 2017). The interview guide was structured to explore the participants’ athletic careers and their life skills throughout their athletic careers, including following key areas: (a) beginning of swimming career (e.g., “How did you get into swimming and when did you start competing? What was your experience joining your first swimming club?”); (b) development and learning throughout their swimming career (e.g., “Throughout your years of training, what do you feel you have learned? Can you share key experiences or lessons gained during your swimming career?”); (c) life skills development (e.g., “What do you think are the most important skills you have learned throughout your swimming career?; What three skills would you place most value on and how important are they for elite swimmers?”); and (d) reflection on career impact and life post-swimming (e.g., “Thinking about your retirement from sport. How do you think having more clarity on life skills makes you feel about your retirement? How different do you think your life would be when it comes to skills if you had not pursued swimming as far as you have done?”). The lead author conducted a pilot interview using the same interview guide. While there were minor adjustments, such as changing the order of the questions, no significant changes were made.

### 2.4 Data analysis and rigor

Thematic analysis (Braun and Clarke, 2006) has been selected for interpreting the data, which is not restricted in the development of themes and codes, thereby offering a more open means to interpret data and the potential for alignment with previous research (Braun and Clarke, 2006; Braun and Clarke, 2019). The procedure started with an in-depth engagement with the data (initial step) and culminated in the articulation of the identified themes (final step). By thoroughly examining the interview transcripts and audio recordings (initial step), preliminary codes were identified, which captured the essence of the participants’ journeys through elite swimming career (second step). To validate and ensure the robustness of our findings, we had regular meetings to discuss the initial codes and the key themes identified from the data (third step). These discussions, carried out via both online and face-to-face meetings, were critical in fine-tuning and reaching a consensus on the themes, thereby ensuring a coherent and unified interpretation of the data. This methodical approach to data analysis was adopted with the intention of building confidence in our results, offering a credible account of the participants’ experiences. In the later phases, to further refine and clarify the findings, both authors thoroughly reviewed, defined, and labeled the themes (fourth and fifth steps). In addition, to ensure the quality of our thematic analysis, we rigorously referred to Braun and Clarke’s (2006) 15-point checklist, applying it throughout the six-step analytical process.

Rose and Johnson (2020) highlighted the critical need to minimize researcher bias. They suggested *member checking* as an effective method to achieve this. Member checking involves compiling and anonymizing the collected data, then sharing it with research participants to verify if it accurately reflects their views on the research question. This process enables researchers to have their findings validated by those directly affected by the results. To ensure the credibility of our study, we followed this approach. The fully anonymized results section of our paper was sent to and confirmed by each respondent for accuracy before finalizing the results section (Rose and Johnson (2020). All research participants reviewed and confirmed the shared results section, and no further amendments were required.

## 3 Results

Two key themes were identified from the thematic analysis: (a) Implicit life skill development through athletic and educational experience, and (b) Understanding the influence of swimming on life skill development. The first theme includes three sub-themes: (a) Establishment of athletic identity, (b) Prioritization of athletic identity, and (c) Navigating life skills through athletic challenges.

### 3.1 Implicit life skill development through athletic and educational experiences

The consensus among participants was that life skills were generally developed unintentionally and implicitly throughout their lives, influenced by both educational and athletic experiences. For instance, P3 shared, “When it comes to life skills, I think throughout my sporting journey it has been mostly subconscious learning. I think moving to boarding school forced me to learn a lot of lessons early in and out of the water… Unconsciously I was learning different skills through studying, swimming, and my downtime.” In a similar sense, P5 reflected on his experiences during his mid-to-late teen years: “I found that you really start to build up a mental resilience and find out what you are capable of. This all happens without you realizing it.” The participants’ narratives frequently reference the implicit acquisition of life skills, either directly or inferred. These skills were developed throughout their careers, shaped by three major factors: (a) Establishment of athletic identity, (b) Prioritization of athletic identity, and (c) Navigating life skills through athletic challenges. These factors are derived from six distinct life experiences (see Table 2). This pattern indicates a common pathway followed by these six participants, primarily through their commitment to excelling in swimming. Notably, all participants acknowledged that there were no specific life skills interventions in their careers, as far as they were aware.

**Table 2:** Factors influencing life skills development.

Factors influencing on life skills development	Associated life experiences
Establishment of athletic identity	Validating self and gaining recognition from others Navigating competitive environments for athletic and life success
Prioritization of athletic identity	Specialization in Swimming Guidance and support from mentors
Navigating life skills through athletic challenges	Balancing dual careers (i.e., sport and study) Recognizing life skills in higher education and post-retirement

#### 3.1.1 Establishment of athletic identity

During the early stages of their careers, without significant results to demonstrate, participants found it necessary to justify their sporting and career choices through early demonstrations of life skills such as work ethic, communication, self-awareness, and teamwork. Five participants shared that despite aspiring to compete in major international championships, they had modest beginnings in their careers, which led to the need to justify their positions to parents, peers, coaches, and themselves. P3 marked, “just trying to gain respect.” P2 needed to prove her intentions to her parents, saying, “I just wanted to prove to them that I wanted to, and I could (do it).” P6, reflecting on a failed trial, said, “I wanted to prove I was good enough….” This need for self and external validation was identified as a common early experience among young swimmers, coinciding with the formation of their foundational athletic identity and the development of life skills such as goal setting and self-drive. It is interesting to note that P6 discussed the impact of his swimming identity on his early personality, noting how it led to him having dual personas – one for school and one for swimming. He viewed this duality negatively, as it hindered his social development at school.

Following the establishment of their athletic identities, participants navigated career transitions and increasingly competitive environments. This progression was crucial for the further development of life skills. As participants moved through more competitive *environments*, their athletic identity and identification with the sport strengthened. P4, describing her experience at the Commonwealth Games, said, “the environment changes you and you learn so much… it gives you a purpose… It always starts with wanting to be the best… Your reality changes and your expectations rise with the occasion.” The term *environment* was frequently mentioned across the participants’ narratives, encompassing various settings such as home swimming clubs, school, performance center, and levels of competition ranging from regional to the Olympic Games. P5, reflecting on his final years of competitive swimming, spoke of finding an “environment that would help reshape me and learn more about how to keep me at the top of my game.” This indicated high self-awareness among all participants, noted as the most prominent life skill. Along with this, their critical thinking and decision-making skills also improved. When reflecting on *the environment*, P3 observed, “we create the environment we want to work in, and we are products of that environment. You want a group of challenging people that are highly motivated and turn up every day chasing high performance. This is something that swimming has changed in me for sure.” P2 also spoke about her experience after participating in her first Commonwealth Games and how it influenced her identity. She described a mindset shift, with new goals set towards the 2021 Olympics: “there was a mindset shift at that point as well… I knew I wanted to go as far as I could with it [swimming] after coming back from the Commonwealth Games.” P3’s reflection highlights how the self-crafted, challenging environment in competitive swimming shapes an athlete’s ambition and performance. Likewise, P2’s experience at the Commonwealth Games illustrates how significant competitive environments can catalyze a profound shift in an athlete’s mindset and goals, marking critical turning points in their sporting journey.

#### 3.1.2 Prioritization of athletic identity

Early in their careers, around mid-teens, all participants started developing self-awareness of their athletic abilities and identities, influencing their self-definition and career paths. P5 realized his swimming talent at about 15 years old, a time when he began to appreciate his work ethic and team support: “All the hard work I had done in the years prior started paying dividends… once I had got a little more confident, I got the hunger for training and competing… 5:30 am training is not a conventional thing for a 12-year-old to do and it was hard work.” For four participants, a critical moment in shaping their athletic identity occurred after qualifying for or competing in their first Commonwealth Games. P4 felt that swimming gave her a purpose, a sentiment echoed by others and influential in defining their athletic careers. The formation of this athletic identity and pursuit of excellence led to the development of key life skills. P3, after the Commonwealth Games, felt “it fully cemented that this is what I love to do,” while P5 saw it as a steppingstone to “chase the Olympic dream.” P4 described major competitions as transformative environments, stating, “…the environment changes you and you learn so much, and it makes you want to keep going… it gives you purpose.” These reflections suggest that swimming competitions are more than just sporting events; they are crucial for identity formation and full immersion in the sport. During the specialization phase, participants needed guidance for direction and final athletic identity formation. This support was psychological such as emotional support from families, support for goal setting. P5 attributes his career success to his supportive environment: “Looking back, I put most of my success down to the environment and the team around me… I would say that I favored the team and the friends I made during that time… it was hard work, but the team and the coaches got me through it.”

Identifying a pathway through higher education was crucial for further developing athletic identity. P4 discussed her transition from junior to senior swimming, coinciding with her move from school to university: “I had good guidance from my coach and parents that some people move quicker than others biologically and that the results very much follow that instead of the work they put in at that age, that really helped me be patient and focus on the things that I could control, which was my work ethic and process goals, things I could control day-to-day.” These career transitions presented rich opportunities for life skill development, although the reporting of these skills did not always reflect the opportunities available. P4 reflected on the broader importance of challenges in life: “more important just to have something in your life than challenges you and it’s more the pursuit of excellence or mastery that teaches those key skills like work ethic.” She questioned whether sport was the only avenue for learning life skills, suggesting that self-challenging situations are key to their development. P2 considered the challenges faced by others: “What do these people find hard? What is hard in your life, where is your limit, do you know? I love the satisfaction of working hard, not floating, taking ownership, and working towards something. I’d love to know what others outside of sport keeps them from floating.” This ties back to the idea that swimming provided a purpose, through which they found challenges and learned life skills. P6 talked about the lessons of ownership and honesty learned through swimming: “I think if you have not done sport, you can develop this idea of victimizing yourself. A really important thing about an individual sport is you have to look at yourself first and ask what could I have done better? At the end of the day, you are the only one in the pool doing it.” He felt that sport prevented him from blaming others for his problems, a tendency he noticed in non-athletes. The participants felt more grounded and decisive in their life choices than their non-athletic peers, highlighting the importance of finding a purpose outside of education, especially during adolescence, for a greater uptake of life skills.

#### 3.1.3 Navigating life skills through athletic challenges

Challenging life skills was identified as a final influential factor in learning before the transfer of skills was possible or achieved, and it was also seen as a crucial aspect of athletic development. Among our participants, five were dual career athletes up to the undergraduate level, while one pursued swimming full-time without other commitments. The development of life skills through sport, which often happens implicitly, can be further enhanced by balancing education with sports. As noted earlier, athletic identity was a primary consideration during schooling, with university choices often influenced by the presence of dedicated performance centers in the UK. This prioritization of swimming influenced how participants managed their academic and athletic commitments.

P3 described the autonomy and ownership gained through balancing school and swimming: “Once I moved to school it was down to me… I knew early on it was down to me… I had to be independent at a young age, stretching myself, just cracking on…Having school and swimming together worked well for setting me up for university as I became really independent.” P4 reflected on how her dual career during school years developed her time-management, discipline, autonomy, work ethic, and positively changed her self-talk during challenging times: “I had to do 2 sessions a week by myself in a more local pool… I had to do my training after I was dropped off then walk to school afterwards, so I started to become more independent around this time, I did not even have a coach during these sessions. I found these sessions quite testing… As I got older, I realized that no one could do the work for me and if I missed it, it was only me that was missing out… it was more positive my self-talk thinking more like I have 30 min left to make a difference.”

P5, who did not initially pursue higher education, reflected on his school years and their enduring impact: “At this stage I found that you really start to build up a mental resilience and find out what you are capable of… Balancing my studies alongside swimming was just something that everyone had to do. Looking back, I realize how hard it was and that it is not an easy thing to do… you just get used to having a lot on your plate… It really helped me after I retired because I started work and went back to university where I was able to manage both well and get a distinction in my MSc. I think the thing that helped me the most on both occasions was being able to look at it and see small steps to achievement the larger goal.” Four participants transitioned directly from secondary to higher education, strategically choosing institutions that supported their swimming careers. P3 noted, “Higher education gave me the challenges I was looking for outside of the pool and I can see now that I have my degree and doing it alongside swimming really helped me.” Participants perceived higher education as a critical arena for learning to manage adversity and uncontrollable life aspects such as relationships or work. P6 summed up this sentiment: “Sometimes you have to grit your teeth, get your head down, and get it done… You need to learn to cope with these challenges in your life because they will always exist or have the potential to exist.” P3’s experience suggests that higher education served not only as an academic pursuit but also as a parallel challenge to athletic training, offering a holistic growth environment. This dual engagement in academia and sports appears to have enhanced the participants’ resilience and adaptability, equipping them with skills to navigate various life challenges, as echoed by P6’s emphasis on perseverance and coping strategies.

### 3.2 Understanding the influence of swimming on life skill development

All participants positively reflected on the influence of swimming in shaping their careers and lives, suggesting that their paths would have been significantly different without it. P5 emphasized learning from non-performance periods, highlighting the importance of handling adversity for career longevity: “I find it’s the times where do not swim a pb [personal best] or do not get to where you want, that’s where you learn the most. Understanding what success and failure looks like and learning from them is key to being able to do as good a job as you can.” Confidence was a recurring skill, with P3 noting, “If I had not pursued swimming as far, I do not think I would be as confident as I am now… [swimming helped] me find purpose and feeling like I belonged.” He believed that swimming also influenced his educational pursuits, contributing to his undergraduate degree attainment. P6 credited swimming for instilling a sense of competence and confidence: “it gave me the confidence I needed to succeed in my life so far and I am sure that it’ll will always help me.”

The ability to translate hard work into progress and *perceived success* was a significant life skill noted by all participants, impacting their life outlook. This led to the development of a strong work ethic. P4 expressed: “Without swimming and pursing it as far as I have, I think I would be lazier… I think having swimming has really helped my work ethic and mental resilience to things… it really helped me at school and University, being able to look inward and competed against myself to be better than I was yesterday has been a great skill that I have learned.” P2 also shared similar sentiments: “I just do not know what I would do without sport, I think without it I would be more likely to take the easy options or rely on others more…, I think I would probably just float through life.” P1, now retired, actively seeks *uncomfortable* situations, mirroring her swimming experiences. She credits swimming with developing emotional intelligence and stress management skills. Participants acknowledged the role of swimming in teaching competitiveness and emotional control, extending beyond the sporting context. P1 shared: “[swimming helped] in terms of managing yourself, nerves etc. [Being able to] stand in front of millions of people without realizing on the tele, that kind of pressure, heightened environment. That has helped me… putting yourself in a vulnerable position which not many people will do.” When asked about potential differences had they not pursued swimming to their current extents, all participants believed they would lack the same level of self-confidence. P3 added that despite being academically capable, swimming was a key motivator in pursuing university education: “I do not think I would be as confident as I am now… I also do not think university would have been for me without swimming. I am not that academic so if I only had university to do, I’m not sure that would have been my thing.” The participants’ reflections indicate that their commitment to swimming was crucial in developing their self-confidence, extending beyond athletic achievements to influence their educational and personal choices. P3’s experience, in particular, highlights how the discipline and self-assurance gained from swimming not only complemented but also compensated for his academic pursuits, suggesting that sports can play a critical role in shaping broader life trajectories and self-perception.

## 4 Discussion

This study aims to explores the experiences of British elite swimmers in developing life skills during and throughout their athletic careers, examining the factors that influence their perspectives on this skill development. This study offers detailed insights into the experiences of British elite swimmers, focusing on the development of life skills throughout their athletic careers and beyond. As a result, these findings enrich the existing literature on life skill development in elite athletes and inform practical approaches for effectively supporting athletes in both developing and transferring these skills.

Life skill development in the elite swimmers, particularly those engaged in both swimming and academics, often occurs in a subconscious and natural manner, rather than through direct and intentional learning. This suggests that environments and experiences, both in educational settings and in sports, play a crucial role in shaping essential life skills. In the initial phases of their careers, when significant achievements were scarce, the participants felt pressure to justify their career choices in swimming. This justification is often manifested through the early display of life skills (e.g., work ethic, communication, self-awareness, and teamwork). It was found in literature that participation in sports nurtures life skills like teamwork, assisting athletes in both their athletic and non-athletic life pursuits (Trottier and Robitaille, 2014). However, the findings from the present study expand on this understanding by demonstrating that athletes develop these life skills under the pressure of having to validate their abilities and career choices in elite sports. Such prevalent early experience among these young swimmers was also the necessity for both self-validation and external affirmation. This period is not just about athletic performance but also about establishing a strong athletic identity and foundational life skills. Individuals possessing a exclusive athletic identity often interpret their experiences from the perspective of an athlete (Lally, 2007), which is supported by the findings in the present study. While previous studies have viewed this single-minded identity and perspective as concerning, as it may hinder the development of other identities and exploration of career options beyond sports (e.g., Kerr and Dacyshyn, 2000; Lally, 2007; Park et al., 2013), the findings in our study suggest that such an athletic identity assists in the development of life skills through sport, which is highly valued by the participants. This provides a new perspective to the existing body of literature.

As they progressed into more competitive environments, the participants’ athletic identity strengthened, leading to a significant increase in self-awareness, identified as a key life skill. These stages of career development, from early justification to navigating competitive challenges, were crucial for holistic life skill development. On the other hand, previous studies have identified various stressors in stressful situations for athletes, such as the competitive environment in elite sports, which may lead to psychological issues such as anxiety (e.g., Hanton et al., 2005, 2009). However, the participants in this study focused more on discussing the positive role of the competitive environment in their life skill development, an aspect that warrants further exploration. This also emphasizes the potential for career assistance programs for high-performance athletes to focus on creating environments that nurture both athletic and personal growth (Torregrossa et al., 2020; Hong and Minikin, 2023), encouraging them to foster life skills that can be applied during and beyond their athletic careers.

The transition to higher education was identified as a critical period for further solidifying their athletic identity and life skill development. Participants felt more confident and decisive in their life choices compared to non-athletic peers, highlighting the value of finding a meaningful pursuit outside of formal education for enhanced life skill acquisition. This suggests the significant value of integrating sports with educational pathways and broader life experiences for comprehensive youth development. Previous studies explored the evolution of athletic career transition research, highlighting that dual career athletes balancing sports with education or work develop valuable personal resources, including dual career management, mental toughness, and adaptability, which are transferable to different life aspects (Stambulova et al., 2021). Lally and Kerr (2005) also suggest that athletes in a dual career setting are more likely to explore non-sporting professions post-retirement, indicating a belief among athletes that life skills acquired in sports are valuable in non-sporting contexts. Aquilina (2013) supports this view and identified benefits leading student athletes to value their dual career, including the development, transferability, and external value of life skills. In the context of the transfer of life skills, as Martinek and Lee (2012) pointed out, there is a gap in our understanding of how life skills are transferred in the context of sports, in particular among high-performance athletes such as Olympians. The findings of this study show that challenging life skills were identified as a crucial factor in athletic development and skill transfer among participants, most of whom balanced their sports careers with academic pursuits. This dual engagement in education and athletics significantly enhanced life skills such as time management, discipline, and positive self-talk. In this regard, higher education was perceived as essential for managing adversity and uncontrollable life aspects. The combination of academic and athletic endeavors was found to significantly strengthen resilience and adaptability in swimmers, preparing them for various life challenges. The findings highlight the value of integrating education with sports in athlete development, suggesting that such a balance can greatly enhance life skill acquisition and overall personal growth. This also emphasizes the importance of educational support (e.g., academic flexibility; English et al., 2022) within athlete support programs and the role of higher education in complementing athletic training. Educational support during the prioritization stage of an individual’s career is critical for assisting with current process goals and guiding the direction of their future career path (Côté and Hancock, 2016). This finding aligns with previous research by de Subijana et al. (2022), showing that athletes with higher education levels at retirement have more advanced individual and social life skills.

Participants highlighted that swimming significantly shaped their careers and personal lives, instilling self-confidence, competence, and a strong work ethic. They credited swimming for positively influencing their educational pursuits and developing essential life skills, such as emotional intelligence and stress management. The sport was seen as key to their success and personal growth, with many expressing that their lives would have been considerably different without their swimming experiences. These findings highlight the significant impact of sport on personal and educational development (Gould et al., 2007; Holt et al., 2008; Trottier and Robitaille, 2014; Pierce et al., 2017). The role of sport in building life skills and self-confidence emphasizes the value of integrating sports into broader developmental programs, suggesting its importance in shaping individuals’ overall life trajectories and self-perception. The acknowledgment by all participants that their careers lacked specific life skills interventions suggests a potential gap in supporting initiatives and services for high-performance athletes. This highlights an opportunity for sports organizations and educational institutions to integrate structured life skills training into their curriculums and programs. Developing explicit programs aimed at enhancing life skills could complement the natural, implicit learning that occurs through sports participation, leading to more well-rounded athlete development (Pummell et al., 2008; Debois et al., 2015; Ryan, 2015). This could also ensure that athletes are better equipped with essential skills for both their sports careers and life after sports. While it has been reported that sport governing bodies and organizations have established career assistance programs for high-performance athletes (e.g., Torregrossa et al., 2020; Hong and Minikin, 2023), our participants’ lack of support in developing and transferring life skills indicates a need for more proactive support and better promotion of such resources to the target population.

Our study, focusing on high-profile elite swimmers in the U.K., provides significant implications and contributions, but it is crucial to recognize its limitations and suggest directions for future research. Although the narratives of six elite swimmers at the Olympic and Commonwealth level were rich and sufficient for answering our research questions, the sample size is relatively small. Future research could benefit from a larger and more diverse sample, such as including more retired swimmers for balance, athletes from different sports, varying levels, and nationalities, to gain broader insights into life skill development and transfer in elite sports. Future studies should also consider focusing on exploring the experiences of retired athletes in transferring their athletic skills to other domains, examining any encountered barriers and coping strategies. This could offer valuable insights into career assistance programs and other athlete support services.

## Data availability statement

The original contributions presented in the study are included in the article/supplementary material, further inquiries can be directed to the corresponding author.

## Ethics statement

The studies involving humans were approved by General University Ethics Panel (GUEP), University of Stirling. The studies were conducted in accordance with the local legislation and institutional requirements. The participants provided their written informed consent to participate in this study.

## Author contributions

RM: Writing – original draft, Project administration, Methodology, Investigation, Formal analysis, Data curation, Conceptualization. HH: Writing – review & editing, Supervision, Methodology, Conceptualization.
